# Large-Scale Phylogenetic Classification of Fungal Chitin Synthases and Identification of a Putative Cell-Wall Metabolism Gene Cluster in Aspergillus Genomes

**DOI:** 10.1371/journal.pone.0104920

**Published:** 2014-08-22

**Authors:** Jose Ramon Pacheco-Arjona, Jorge Humberto Ramirez-Prado

**Affiliations:** Unidad de Biotecnologia, Centro de Investigacion Cientifica de Yucatan, A.C., Merida, Yucatan, Mexico; Seoul National University, Korea, Republic of

## Abstract

The cell wall is a protective and versatile structure distributed in all fungi. The component responsible for its rigidity is chitin, a product of chitin synthase (Chsp) enzymes. There are seven classes of chitin synthase genes (*CHS*) and the amount and type encoded in fungal genomes varies considerably from one species to another. Previous Chsp sequence analyses focused on their study as individual units, regardless of genomic context. The identification of blocks of conserved genes between genomes can provide important clues about the interactions and localization of chitin synthases. On the present study, we carried out an *in silico* search of all putative Chsp encoded in 54 full fungal genomes, encompassing 21 orders from five phyla. Phylogenetic studies of these Chsp were able to confidently classify 347 out of the 369 Chsp identified (94%). Patterns in the distribution of Chsp related to taxonomy were identified, the most prominent being related to the type of fungal growth. More importantly, a synteny analysis for genomic blocks centered on class IV Chsp (the most abundant and widely distributed Chsp class) identified a putative cell wall metabolism gene cluster in members of the genus Aspergillus, the first such association reported for any fungal genome.

## Introduction

The fungal cell wall is a protective structural complex that controls permeability, protects the cell against osmotic changes, and shapes it. It is typically composed of interconnected polysaccharides such as chitin, (1,3)-β-glucan and (1,6)-β-glucan, mannan, and proteins [1]. Chitin synthase enzymes (Chsp) manufacture large linear chains of β-(1,4)-linked N-acetylglucosamine. Within most fungi chitin is the component that gives rigidity to the wall; mutations that eliminate the ability to synthesize chitin can be lethal to fungal cells [2].

Chsp protein sequences can be very diverse and show varying combinations of domains, but several studies agree that all—from both yeast and filamentous fungi— contain three conserved motifs: QXXEY, EDRXL, and QXRRW [3]–[6]. These conserved motifs in all Chsp are restricted to a short stretch of peptide called the Conserved Region 1 (CON1) [7], which is the core of the Chitin synthase 2 domain CS2 (Chitin_synth_2; PF03142). The EDRXL motif is related to the catalytic base function; QXRRW (QXRRWXN in Nagahashi et al. [7]) confers processivity to the enzyme; and QXXEY does not have a putative function assigned [4].

The various Chsp sequences have been classified phylogenetically into seven classes (I–VII) grouped in two divisions [4]–[6]. Classes IV–VII contain the CS2 domain while Chsp from classes I–III have lost the first portion of CS2, replacing it with both a type 1 Chitin synthase domain CS1 (Chitin_synth_1; PF01644) as well as a Chitin synthase N-terminal domain CSN (Chitin_synth_N; PF08407), but preserving the CON1 region from CS2 [6]. In addition to these domains, class I Chsp have conserved motifs present in the amino terminal portion; classes IV, V, and VII contain a binding domain similar to cytochrome b5 (cyt-b5; PF00173); and classes V and VII also contain a myosin motor domain (Myosin_head; PF00063) [6]. The class VI Chsp domain structure is the simplest with only a CS2 domain and could be the ancestral state of Chsp [6]. For simplicity, hereafter the Chsp classes will be labeled as ChspI to ChspVII. The use of different methods of phylogenetic inference, among other factors, has caused multiple Chsp classifications, which becomes a problem when comparing results from independent studies. The two main classifications, from Choquer et al. [4] and Mandel et al. [6], concur in grouping Chsp in two divisions in which ChspI-III belong to division 1, ChspIV, V, and VII belong to division 2, and ChspVI is on its own outside both divisions. However, their nomenclature for ChspVI and VII disagrees: Mandels's ChspVI is Choquer's ChspVII and vice versa. Mandel's ChspVI nomenclature is based on the description of *Aspergillus fumigatus* class VI AfChsD [8], which precedes Choquer's and is the one used on this study. Another study, by Odenbach et al. [9], positions ChspVII (actually Mandel's ChspVI) inside division 1, as a sister clade of ChspI-III. In a more recent study Ruiz-Herrera and Ortiz-Castellanos [10] speculate that ChspIV is actually the common ancestor of all fungal CHS and that the other classes would have evolved by events of duplication and modification.

The availability of full fungal genome sequences allows us to look into the evolution of Chsp from a new perspective, not as individual gene units but as part of a genomic neighborhood or syntenic group. In bacterial genomes, the syntenic blocks are usually organized as operons [11]. The syntenic blocks in eukaryotic genomes are much more complex; the evidence suggests that these may form different types of functional aggregations [12] and topological arrangements [13]. In both prokaryotic and eukaryotic genomes, as the evolutionary distance between species increases, the syntenic blocks rearrange or break up. This can be seen, in a large scale, in the comparative study between chromosome 12 of *Mycosphaerella graminicola* and scaffold 7 of *Stagonospora nodorum*, both of the class Dothideomycetes [14]. It was observed that the orientation of orthologous genes was arranged randomly, but in a genomic context there is not a random distribution. This would imply conservation in gene content between these genomic units during molecular evolution. Even in closely related species of the same genus when there is a strong pressure, as is the case in pathogenic systems, recent changes in synteny conservation can be observed. This was the case for the comparative genomics study of the whole genome of the highly pathogenic *Candida albicans* versus its closest relative, *C. dublinensis*, a significantly less virulent pathogen [15].

The above evidence indicates that strong selection pressures act to maintain areas that retain genes in close order and that these may form several types of functional groups. We believe that the analysis, on a large scale, of the distribution of Chsp among diverse fungal species can provide important molecular evolution information. Due to its importance in cell-wall metabolism, some chitin synthases may be forming genetic clusters with functionally related genes as a manner of coordinated regulation. To test this hypothesis, we first systematically searched for all the putative Chsp present in 54 fungal genomes. The 369 Chsp identified were classified by five methods of phylogenetic inference. From this classification, an analysis of the qualitative and quantitative distribution of Chsp among the individual fungal species was derived as well as a general analysis of the distribution and organization of Chsp in all the species (a relationship is observed between the content of Chsp and the taxonomy and growth form of the fungus). This distribution analysis showed that ChspIV is the class with the largest presence in the fungal species, and as such it was selected to conduct synteny analysis searching for groups of genes functionally related to the biogenesis of the cell wall, revealing an association between at least six genes in members of the genus Aspergillus.

## Materials and Methods

### Fungal genomes

For this study, a collection of the protein models for 54 fungal genomes (Table S1) was used. We relied on the genome annotations provided by the corresponding sequencing project. The data were taken from: Fungal Genome Initiative-BROAD Institute (BROAD- FGI), Department of Energy Joint Genome Institute (JGI), National Center for Biotechnology Information (NCBI), The Institute for Genomic Research (TIGR), Wellcome Trust Sanger Institute, Genomic Exploration of the Hemiascomycete Yeasts (Génolevures), and the National Institute of Technology and Evaluation (NITE).

### Putative Chsp search

A search for putative Chsp was performed on the protein models corresponding to the 54 fungal genomes. For this, a regular expression (RE) based on the shorter version of the CON1 region (hereafter called CON1S) containing the three conserved Chsp motifs QXXEY, QXRRW and EDRXL [3], [4], [6], was used: “Q‥EY[A-Z]*EDR.L[A-Z]*Q.RRW” (periods represent a single amino acid). The RE was coded in a script written in the Perl programming language [16] (File S1).

The sequences retrieved with the RE were then used to generate a profile for a hidden Markov model (HMM) search [17] with the HMMER 2.3.2 [18] package. A multiple alignment of 369 putative Chsp was performed with the program ProbCons [19]. The HMM search was run on the original protein models of the 54 fungal genomes with a per-sequence E-value cutoff of < = 1e-23. All sequences recovered from the RE search as well as the HMM search were scanned against PfamA InterPro's signatures [20] to detect probable domains using Interpro pluging for the program Geneious 6.0 [21].

### Phylogenetic inference I: Canonical conserved motifs of Chsp

The 369 putative Chsp sequences found in this work (Table S2), plus 49 Chsp retrieved from the UniProt database [22] (Table S3), were trimmed to only include the region encompassed by the QXXEY, QXRRW, and EDRXL motifs (CON1S). The resulting fragments were used to generate a multiple alignment with the MUSCLE algorithm [23]. The obtained alignments were edited to preserve the parsimony informative sites. Phylogenies based on this multiple alignment were inferred by five different methods. Chsp sequences from insects were included as outgroup: *Drosophila melanogaster* chs1 AAG09735, *Lucilia cuprina* chs1 AAG09712, *Aedes aegypti* chs XP_001651163, and *Dirofilaria immitis* chs AAL92023 (Table S3).

Phylogenies constructed by minimum evolution (ME), neighbor joining (NJ), and maximum parsimony (MP) methods were conducted with MEGA5 [24]; for ME and NJ a model based on the JTT matrices [25] (in units of number of substituted amino acids per site) was used. Trees inferred by ME and MP methods were carried out using the Close-Neighbor-Interchange algorithm [26]. A phylogeny by the Maximum Likelihood (ML) method was performed with PhyML [27] based on the evolutionary model LG [26] and with a gamma distribution of 0.703; both the model and gamma parameter were determined with the ProtTest3 program [28]. The search for the topology was conducted with Best NNI and SPR. Finally, a tree inferred by the Bayesian method was performed with MrBayes using its plugin for the program Geneious 6.0 [21] based on the WAG evolutionary model [29] with both a gamma distribution and a proportion of invariable sites uniformly distributed in the intervals (0.00, 200.00) and (0.00, 1.00), respectively. The evolutionary model was determined with MEGA5. A variation rate among sites was used with four categories. The length of chain was 1.1 million with a 200 sample frequency.

### Phylogenetic inference II: Full Chsp protein sequences

A multiple alignment of the complete protein sequences of the 369 putative Chsp found in this study was performed using the MAFFT [30] program. The alignment also included the full protein sequences of 34 Chsp retrieved from Uniprot [22] and outgroup sequences were used as above (Table S4). Two phylogenies were inferred employing the neighbor joining (NJ) and Bayesian methods. The NJ method was performed under the Geneious [21] program using the Jukes-Cantor model [31]. There were a total of 3695 positions in the final dataset. The Bayesian method was performed with the MrBayes 3.2 program [32] and was based on the WAG evolutionary model [29] with both a gamma distribution and a proportion of invariable sites uniformly distributed in the intervals (0.00, 200.00) and (0.00, 1.00), respectively. The posterior probability of the approximate tree was found with the numerical Markov Chain Monte Carlo method [17] with a one million chain length and 500-sample frequency.

### Chsp classification by phylogeny consistency

The putative Chsp were classified according to the results obtained from the phylogenetic inferences I and II. For this purpose, resolved monophyletic clades were identified for each method and probable classes were assigned to each clade (based on the Chsp sequences retrieved from Uniprot). Subsequently, clades of the same class but from trees obtained by different inference methods were compared. Putative Chsp were thus assigned to a particular class if found in at least three of the five methods of the phylogenetic inference I. Putative Chsp that did not meet any of these criteria were marked as “unclassified.” Some already reported Chsp were not found due to being located on poorly assembled genomic regions and were manually added and marked as “missing.”

### Distribution of putative Chsp on fungal genomes

Once classified, putative Chsp were quantified and qualified according to species. The data obtained was fit to a species tree of fungi (modified from [33]), indicating the identified copy number of each putative Chsp class present on the genomes of the selected species. Chsp not grouped on clades were labeled as “unclassified Chsp” and those previously reported but not found on this study were labeled as “missing Chsp.”

### Gene cluster identification by synteny analysis

The genomic neighborhood of ChspIV was selected for synteny analysis since it is the most widely distributed class among the fungal species studied. Initially, a phylogeny of all putative ChspIV protein models was obtained. For this, a multiple alignment of 81 ChspIV sequences (Table S5) was done with the MAFFT [30] program. The alignment was edited to preserve the conserved sites between the QXXEY, QXRRW, and EDRXL regions. Phylogenetic trees were constructed using five methods: NJ, ME, MP, ML, and Bayesian inference. The methods were performed in the same way as indicated in the Phylogenetic inference I section, with the difference that the ML method used a gamma distribution of 0.506.

Using the genome displayer of the Geneious 6.0 program [21], individual putative ChspIV genes were located on each genome. Genomic block fragments including ChspIV and neighboring genes (15 upstream and 15 downstream) were extracted. The genomic blocks were divided in “syntenic groups” for analysis following the same order of the clades formed by the ChspIV phylogenies.

Syntenic analysis was performed by alignment of the genomic blocks with the program Mauve [34] to find orthologous regions free from genome rearrangements (Locally Collinear Blocks or LCBs). Predicted genes encoded on the LCBs were analyzed through Blast2GO [35] to assign them putative functional annotations. Recursive TBLASTX searches [36] were carried out between predicted genes of different “syntenic groups” to test the hypothesis of extended synteny through the clades.

To test for probable expression correlation of the members of the putative cell-wall metabolism gene cluster, we used publicly available transcriptomics data for *Aspergillus oryzae* [37]. We obtained the raw CEL files from NCBI GEO (GSE9298) and all chips where processed and normalized with the R:Bioconductor package “affy” [38] using RMA [39]. We assessed correlation and clustering using Kendall's coefficient of concordance implemented in the R statistical package.

## Results and Discussion

### Putative Chsp search

Since not a single domain is common to all fungal Chsp, to massively identify all putative sequences regardless of class, a script was developed that employs a regular expression (RE) designed from the three universal motifs present in the CON1S region. The script identified 369 putative Chsp sequences (Table S2) from 54 fungal genomes representing 21 orders grouped in five phyla. An InterProScan search (against the PfamA database) identified a CS2 domain in 198 of these sequences while 152 sequences had a combination of the CS1 and CSN domains. The remaining 19 sequences contained the three domains CS1, CS2, and CSN (Figure S1). The identification of known CS domains in addition to the presence of the CON1S motifs suggest that most of these sequences are functional Chsp. Nagahashi et al. [7] determined, by site-directed mutagenesis at the CON1 region, that even conserved changes of most of the amino acids in the three universal motifs resulted in complete or almost complete loss of its activity and that only a few conserved changes could maintain the function. To find plausible functional variants of the CON1S region, we used the 369 sequences as a training set for an HMM search. All 369 starting sequences were recovered, with e-values ranging from 1.7e-29 to 1.9e-76. With this approach, an additional 22 putative Chsp were found (Table S6) with e-values ranging from 2.10e-26 to 1.90e-68, and chitin synthase domains for these sequences were detected by an InterProScan search (Figure S2). However, 12 of these sequences completely lacked one of the three conserved domains: seven the QXXEY motif, one the EDRXL motif, and four the QXRRW motif. Another nine had non-conserved amino acid substitutions in one or more of the residues presumed to be critical for activity. Only one out of these additional 22 protein models, t_52578 of *Fusarium solani*, contains the complete three conserved motifs with plausible conserved amino acid substitutions in the EDRXL (DDRVI) motif.

The two observed changes are strongly conserved substitutions: E to D (both are acidic and polar charged; aspartic acid differs from glutamic acid only in that its side chain is shorter by one methylene group); and L to I (isoleucine being an isomer of leucine is also aliphatic, hydrophobic, and non-polar). A mutant generated by Nagahashi et al. [7] harboring the E to D substitution seen here did retain activity. An InterProScan search detected a CS1 domain for this sequence. High Scoring Pairs (HSP) to ChspIII members, with E-values = 0, were obtained when using this sequence as a BLAST query against NCBI's genbank database. All of the above suggests that it could be a functional Chsp with a variant CON1S region, making it an interesting candidate for future experimental confirmation. For the rest of this study only the 369 sequences retrieved by the RE were used to avoid adding complexity to the CON1S region. These results make a point that using only domain prediction to identify putative Chsp produces a high rate of false-positives if the presence of the three universal motifs is not also enforced: although the CON1S region is “the core” of the CS2 domain InterProScan reports a CS2 domain for 17 of the 21 sequences that have truncated versions of this region. Also, domain prediction alone can only determine at best the division to which the putative Chsp belongs since some classes share the same domain architecture (e.g., ChspI-III or ChsV and VII, Figure S3).

### Phylogenetic inference I: canonical conserved Chsp motifs

As a way of massively assigning the most likely class to each of the 369 putative Chsp found, phylogenetic inferences of their CON1S region, including the guide Chsp sequences already classified, were constructed. The length of the CON1S region for the 369 sequences ranges from 104 to 148 amino acids residues (of which 87 to 112 are parsimoniously informative). Five different methods (based on distance as well as on discrete characters) were used to account for possible clade discrepancies due to the high variability of the divergent sites. As expected there are some differences in the topological conformation of trees between methods. These differences did not alter the resolution of clades, as shown by how the guide Chsp are consistently grouped in each method. The five inference methods reconstructed two divisions as expected, but with some differences in the clades that comprise them.

Clades of trees inferred by distance methods [NJ (Figure S4) and ME (Figure S5)], are resolved in a similar manner (Table S7) grouping matching Chsp, with the exception of the polytomy formed in the groups identified as ChspV, VII, and a group of Chytridiomycota in the NJ method. Of particular note is the fact that these trees include ChspIV as part of division 1, contrary to all other classifications.

**Figure 1:**
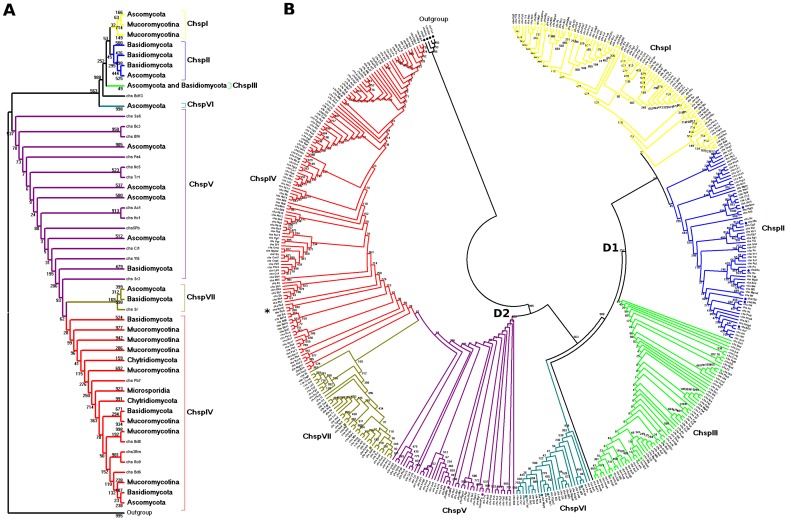
Phylogenetic analysis of amino acid sequences of a partial sequence of Chsp. Tree inferred from conserved motifs (CON1S) with the ML method using the evolutionary model LG. Bootstrap consensus tree, inferred from 1000 replicates, to represent the evolutionary history of the taxa analyzed. (A) Condensed tree showing topology of the classes' distribution among the analyzed phyla. (B) Circular topology representation including all sequences identified.

The discrete characters methods [ML (Figure 1) and Bayesian (Figure S6)] resolve clade ChspVI as part of Division 1 (Table S7), contrary to the ME method, which presents this same clade as an independent group from both divisions. The tree inferred with the Maximum Parsimony (MP) method (Figure S7) resolved the Chsp groupings in a similar way to the distance methods (Table S7), except for the location of the ChspIV clade, which is shown as part of Division 2 along with clades ChspV and ChspVII, consistent with the inferences by the discrete characters methods (Table S7). This method also suggests that clade ChspVI is a sister clade of ChspVII, a feature not seen in other methods.

All distance methods, as well as the MP method grouped ChspIV sequences from all five Phyla analyzed.

The average number of sequences per class for the five methods were ChspI = 57 (SD = 4.63), ChspII = 67.4 (SD = 9.18), ChspIII = 56.2 (SD = 2.17), ChspIV = 85.6 (SD = 3.13), ChspV = 49.6 (SD = 10.06), ChspVI = 25 (SD = 0.71), and ChspVII = 40.2 (SD = 1.92).

### Phylogenetic inference II: Full Chsp protein sequences

A second set of phylogenetic inferences was carried out using the full Chsp protein sequences. The presence of specific domains and particular regions on the complete sequences may increase the information available for the classification, but also increases the complexity of the tree reconstruction due to the high diversity between the classes. Only a distance method (NJ) and a discrete characters method (Bayesian) were used due to the highly variable nature of the full sequences. The tree inferred with the NJ method (Figure S8) shows two divisions; Division 1 is composed of clades ChspI-III and VI, while Division 2 is constituted by the ChspIV, V, and VII clades. The tree inferred with the Bayesian method (Figure S9) also groups clades ChspI-III on Division 1, but includes ChspVI as a sister clade of ChspIV under Division 2 (Table S8).

There is a polytomy between clusters belonging to ChspI and II classes in the NJ inferred tree; however, these can be differentiated by the guide Chsp. Table S8 summarizes the clades and subclades that comprise each division for each method as well as the number of Phyla for the clades.

### Chsp classification by phylogeny consistency

Even though there are differences in how the methods associate the clades on the expected divisions, comparing the individual sequences in each clade it was possible to assign with confidence putative classes to 347 out of the 369 Chsp (94%) (Table S9). The phyolgenetic inference I (using only the CON1S region) showed a higher consistency on the grouping of Chsp on putative classes in comparison to the phyolgenetic inference II (using the full Chsp sequences). When considering selection criteria of the presence in three out of the five inference methods, an average of 89% of the sequences can be consistently assigned to the same class/clade: ChsI 87%, ChsII 84%, ChsIII 93%, ChsIV 100%, ChsV 82.5%, ChsVI 96%, ChsVII 79%. In contrast, the average number of sequences consistently assigned to classes using the full sequences and a criteria of presence in both methods used (NJ and Bayesian) was only 70%: ChsI 54%, ChsII 54%, ChsIII 100%, ChsIV 66%, ChsV 71%, ChsVI 83%, ChsVII 62%. If the same criterion (presence in NJ and Bayesian) is applied to the inferences constructed from the conserved motifs region, this region outperforms the consistency percentage of the full sequence: 90% (ChsI 82.5%, ChsII 80%, ChsIII 100%, ChsIV 94%, ChsV 90%, ChsVI 96%, ChsVII 88%).

Ruiz-Herrera and Ortiz-Castellanos [10] conducted a phylogenetic inference by NJ proposing two divisions with only five Chsp classes but with multiple subclades. Division 2 contains classes IV and V, the latter formed by seven subclades: Basidio Va, Asco Va, Chytridio V, Basidio Vb, Asco Vb, Mucoro Va, and Mucoro Vb. The first three subclades correspond in our study with ChspVII three subclades, which group the same phyla (Figure S5); the next three subclades correspond to ChspV. Their subclade Mucoro Vb in our study corresponds to a group of unclassified Chsp composed exclusively by Mucoromycotina (marked with an asterisk in Figure 1 and Figures S4 to S9). Depending on the used method (distance/characters) and data set (CON1S/full sequence) these sequences are associated to either division 1 or 2. This could be an independent class exclusive to Mucoromycotina, originated from a modified class V.

Although the class-specific domains (e.g., myosin-head-like or cytochrome b5-like) could help discern between Chsp of Division 1 and 2, the highly variable inter-domain regions are difficult to assign to homologous positions in the multiple alignments greatly confounding the inference reconstruction. The domains present in each of the 347 classified were also checked, and corresponded to the expected domain architectures for each class (Figures S1 and S2). Interestingly, the 19 sequences that contained the three CS domains (the CS1-CSN combination plus the CS2 domain) were phylogenetically classified as ChspI, ChspII, or ChspIII, and could represent an intermediate stage before the loss of the first portion of the CS2 domain [6]. Altogether, the variable sites (parsimony-informative) present in the CON1S region provide enough information (either as distance units or discrete characters) to confidently discern between the seven Chsp classes despite the lack of specific domains. The topology of individual classes/clades is better resolved by distance methods, but for the association of classes to their known divisions character methods are more reliable. For example, this is the case for ChspIV, which is assigned to division 1 by the ME and NJ methods when using only the CON1S region, and to division 2 on the trees inferred by the ML, MP, and Bayesian methods.

Due to the shared domain architecture for some of the classes, it is not feasible to design Hidden Markov Models capable of discerning between such classes. Classification by phylogeny, as we have shown, has a high degree of accuracy but can be computationally expensive. As a faster, simpler method of automatic classification, here we propose a series of “Chsp Regular Expressions” (ChspRE) specific for each class. Building on the data carefully classified in the present study—347 sequences confidently classified—we systematically determined the minimum sets of amino acids residues combinations unique for each class (Table 1). The use of the proposed ChspRE is made in an iterative fashion. Firstly, the original RE is used, retrieving Chsp sequences regardless of class but ensuring the CON1S region is present; secondly, the class-specific ChspRE is used to recover only Chsp sequences from that particular class. For some classes, more than one ChspRE can be used (e.g., eight for ChspIII). No ChspRE specific for ChspI or ChspV could be determined, but identification can be done by first recovering sequences from the appropriate division, followed by discarding the “sister classes.”

**Table 1:** Chsp Class specific Regular Expressions.

Class	ChspRE^a^
Class II	R.{5,7}R‥[E|Q].{3,9}E.{6}RT…[V|I][M|Q].N…[L|F]C…….W.{4,5}W.[K|T][I|V].[V|I]‥[V|I][A|S]DGR…….L‥[L|I]…G.{14,18}AH‥E.{17,22}P.Q‥FC.KE.N‥K[L|I]NSHRW.[F|L].[A|S]F…[L|I].P.[V|I]‥[L|M].DVGT.[P|L].{5}Y.LW[K|R].[F|M].{5}[V|I].G[A|V][A|C]G[E|Q].{4,6}G.{7,9}NPL
Class III	RY[T|S]A.TCDP[N|D][E|D]F…[N|A]G‥L[R|K]….[N|G][R|N].T[E|D][L|I]L[I|V][A|C][I|V]T.YNE[D|N]K.L‥RT[L|M]H‥M.N
	W‥I.[V|C].[L|F][V|I].DG….D
	T[T|S]‥S.{6}L‥P.{7,10}L.PV[Q|S]‥[F|L][C|V].K‥N.KKINSHRWL[F|L][N|T][A|G].{4}L.P[E|D]…L[L|I]DAGTKP
	NPLVA.QNFEYK.SN[I|V]LDKP.ES.FGYV.VLPGAFS[A|C]YR[F|Y].A[I|L].G.PL.QYFHGD[H|A][T|S]L
	NMFLAEDRILCFEL.[A|V]K….W.L.Y[V|I][K|R]‥[K|R][G|A]ETDVPE
	E…QRRRWLNGSFA
	W.{8}T…I‥[L|I][A-Z]*GN[R|K]PK
	G.{4}[A|G]…[T|I].G.[Y|N]…[S|A]‥Y.DPWH…S.{12}N[I|V]L[M|N][V|I]YAF.N[W|L]HDVSWGTKG[S|A]D
Class IV	DADT.{23}CGET.I.NK‥[S|T][W|F].[T|S].[I|M]QV[F|Y]EY[F|Y].SH[H|N]‥K.FE‥[F|L]G
	P.{10}Y.{6}TLH.KNL[L|Y][L|H]LGEDR[Y|F]L[S|T].{4,6}F[P|Y].R…[F|Y]…A.C.T.[V|A]P.{5}L.SQRRRWINST.HN[L|M].[E|D]L.{6}CG‥C.S
Class VI	THL.M
	LF[I|V]DSD[C|I]IL
Class VII	G[K|R].[S|T]E.{2,4}KPGNR[G|E]KRD[S|T]Q…‥[F|Y][L|F][N|S][R|K]V…‥M.P[L|M]EL[E|D].[F|Y].[Q|H]‥[N|D][V|I]IG[V|I]DP‥YE[Y|F]…[V|I]DADT.V‥[D|E][S|A]LN
	GICGET.L.N[E|D]‥[T|S].[W|T]TM[I|V]Q[V|I]YEY[Y|F]ISHH[L|M].K[A|S]FE[S|W]LFGS[V|L][T|S].?[L|R]P[G|S].?F‥YR[L|I].{2,9}[I|L]I.{7}Y
	TLH.KNL[L|F][S|A]LGEDR[Y|F]LTT[L|I][M|L].[K|R].[F|W]P.[M|F]‥[K|T]F…A…[T|M].A[P|H]….[V|I]L.SQRRRWINST[I|V]HNL
Division 1	K‥N.[K|R]K[I|L]NSH[R|L]W.[F|L]……‥P….[L|M].D.GT
	P[L|M]‥YF.[G|C]
Division 2	SH…K.FE
	TLH‥NL

a ChspRE spanning multiple lines should be treated as a single string.

### Distribution of putative Chsp in fungal genomes

**Figure 2:**
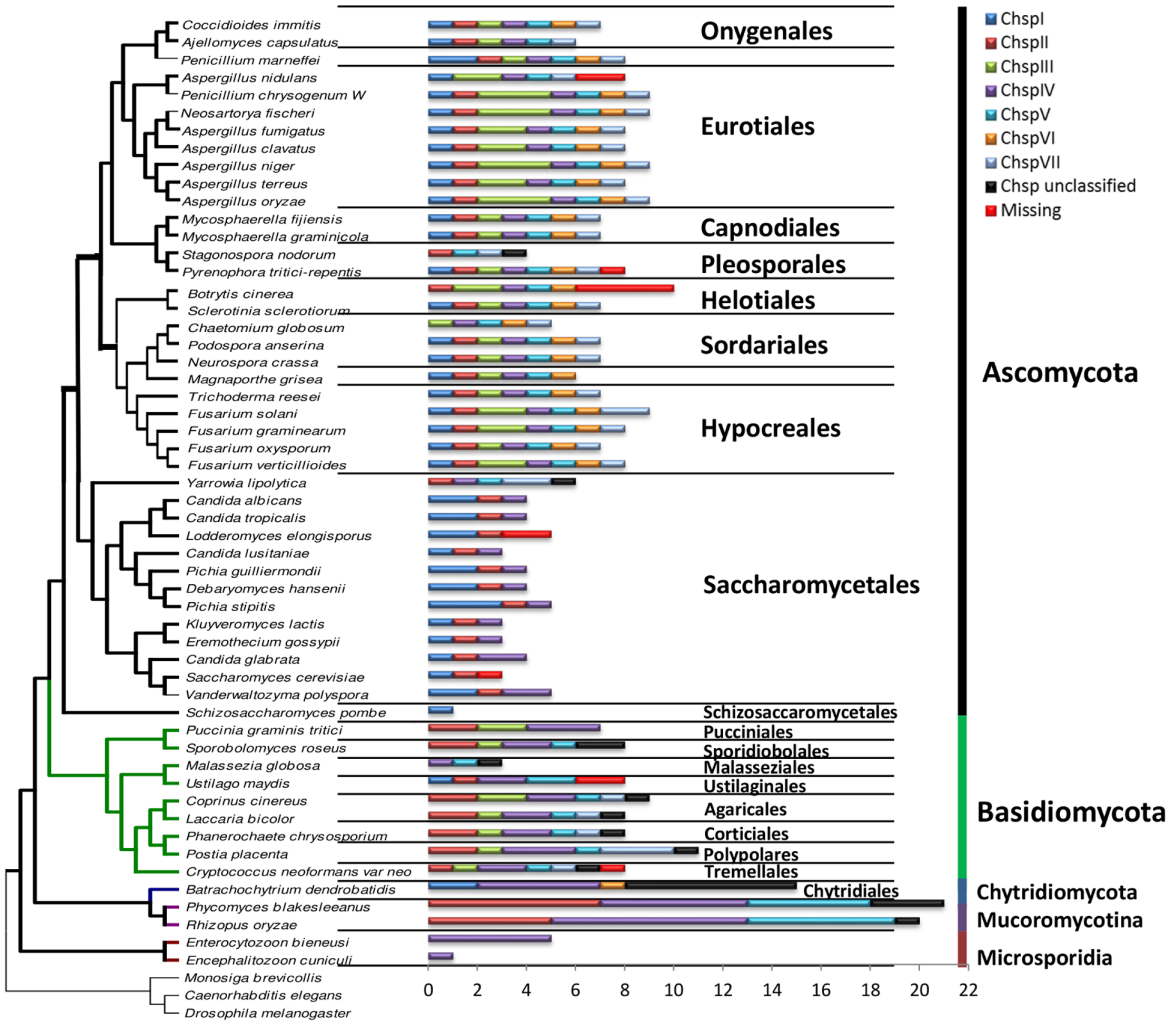
Chsp distribution in fungal species diagram. Phylogeny of 54 species of fungi. Bars indicate the class and copy number of putative Chsp in the selected genomes. Chsp previously reported but not found on this study are labeled as “missing.” Phylogeny tree modified from [33].

The quantity and class of Chsp genes encoded in a particular fungal genome can vary greatly; from a single gene, multiple copies of just a couple of classes, to one (or more) copies of every single class. Genes encoding synthases belonging to ChspIII, V, VI, and VII are found only in fungi with high contents of chitin in their cell walls [40], while filamentous fungi can contain up to ten CHS isoenzymes disseminated among all classes of the two divisions [4]. The 347 putative Chsp sequences reported here show a marked distribution pattern across the different orders and phyla (Figure 2). The most abundant and widely distributed Chsp class corresponds to ChspIV, whose 81 sequences were found in 50 of the 54 analyzed genomes, representing the 21 orders and five phyla analyzed. This wide distribution supports the hypothetical scheme of Ruiz-Herrera and Ortiz-Castellanos [10], with ChspIV as the common ancestor of fungal CHS, latter giving raise to all other classes. This evolutionary history is also supported by all our phylogenetic inferences since the closest non-fungal Chsp (from insects) are consistently positioned outside the divisions, regardless of reconstruction method. Conversely, ChspVI was the least abundant, present as single copy in just 23 of the Ascomycota genomes and the only Chytridiomycota analyzed. Although this distribution of ChspVI could point to an acquisition by horizontal gene transfer, we weren't able to detect such a genomic signature.

Filamentous fungi of the Ascomycota order show the greatest diversity of Chsp classes present in their genomes: 20 of the 26 studied species have at least one copy of every Chsp class. Species belonging to the Eurotiomycetes class contain in their genome the seven Chsp classes, except for *Ajellomyces capsulatus* and *Aspergillus nidulans*. At the level of Order, the Eurotiales and Onygenales differ in that the former feature two to three copies of the ChspIII class whereas the Onygenales only have one copy (Figure 2). Members of the Capnodiales order own a copy of each class. The two species from the Pleosporales order differ greatly in Chsp content: *S. nodorum* only has four genes (ChspII, V, VII, and an unclassified) in contrast to all seven classes present in *Pyrenophora tritici-repentis*. Sordariales *Podospora anserina* and *Neurospora crassa* contain a copy of each class Chsp while *Chaetomium globosum* only has the ChspIII -VII classes (Figure 2).

The Hypocreales order has seven classes, with the characteristic that *F. solani*, *F. graminearum*, and *F. verticillioides* have two copies of the ChspIII class. More than 80% of the species of the Saccharomycetales order, which grow as yeast, contain the ChspI, II, and IV classes. For the Ascomycota, ChspIII, V, and VII classes are only found in filamentous fungi and some dimorphic fungi. *Schizosaccharomyces pombe* is the only Ascomycota which has only one Chsp sequence—a copy of the ChspI class. All of the species of the phylum Basidiomycota lack class ChspVI. The Pucciniomycotina subdivision did not present a recognizable pattern of distribution. The species of the Subphylum Ustilaginomycotina grow as yeast and do not contain any of the ChspIII, VI, and VII classes. The Agaricales, Corticiales, Polypolares, and Tremellales orders possess the ChspII-V classes as well as the VII, with the exception of *Cryptococcus neoformans* var. *neoformans* which lacks class II. The Mucoromycotina subphylum only possesses Chsp from classes II, IV, and V but with multiple copies of each class. The Phylum Microsporidia contains only the class ChspIV (Figure 2).

### Cell-wall metabolism gene clustering

To test our hypothesis that CHS genes are not randomly arranged in fungal chromosomes but actually associated to functionally related genes in a cluster-like manner, we selected CHS genes from class IV (ChspIV) to analyze their genomic neighborhood for functionally related genes (especially cell-wall metabolism genes) as well as syntenic conservation across different orders. It can be easily noted on the distribution analysis that this particular class of Chsp is the most abundant and widely distributed among the diverse phyla of fungal species. In *Saccharomyces cerevisiae*, Chs3 (a class IV Chs) is responsible for the synthesis of 90% of its chitin content [40].

**Figure 3:**
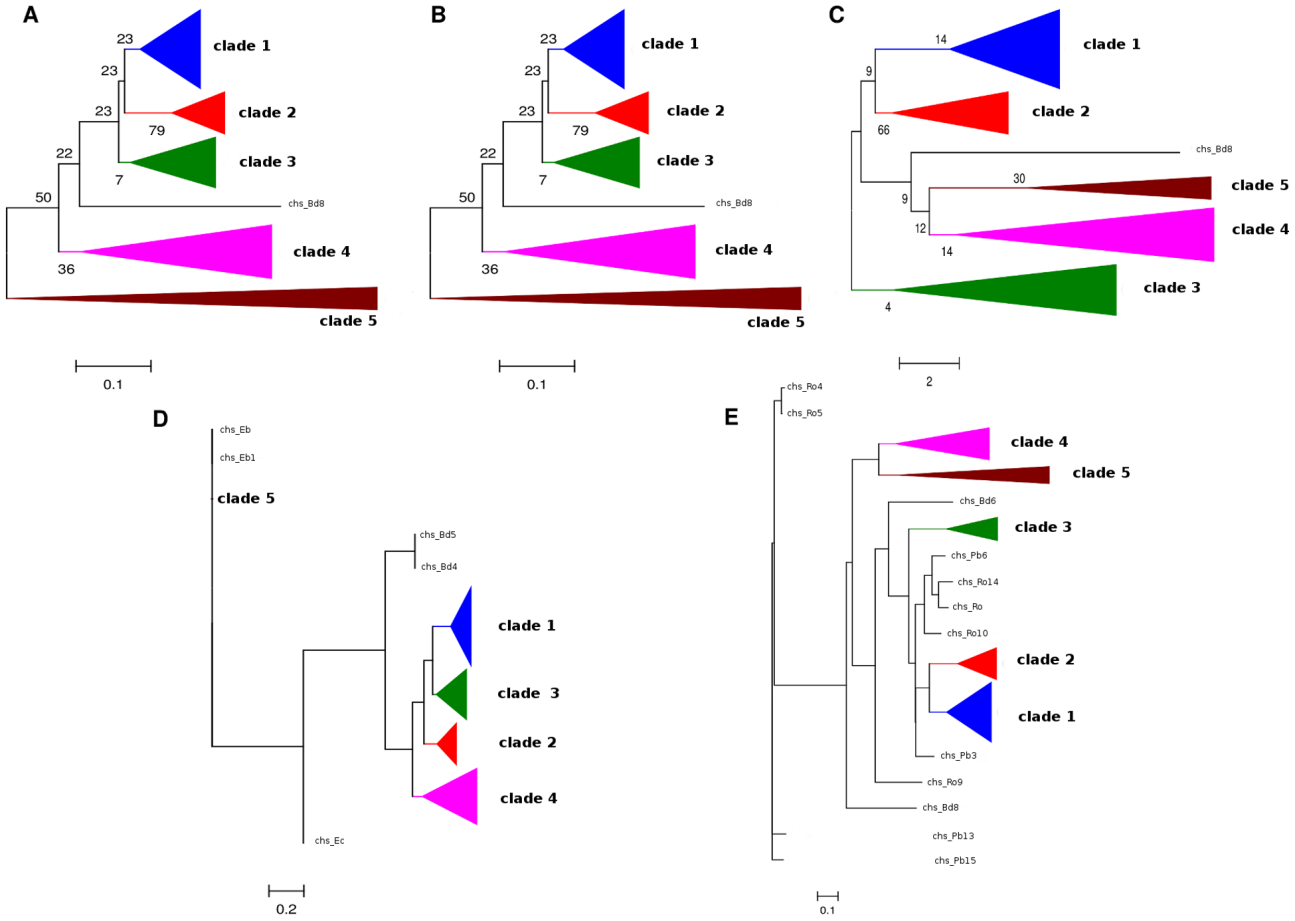
ChspIV phylogenies. Phylogenetic trees inferred using the methods: (A) ME, (B) NJ, (C) MP, (D) ML, and (E) Bayesian. The triangles of the same color represent groupings of the same putative Chsp sequences.

The 81 sequences putatively identified as belonging to the ChspIV class were used to reconstruct phylogenetic inferences for selection of probable syntenic groups. Figure 3 shows the five phylogenetic trees reconstructed from a CON1S region alignment. Even though the arrangement of clades varied depending on the inference method used, every method resolved the same five clades, and each clade contained the same sequences. The blue clade (clade 1) groups Ascomycota filamentous fungi; the red clade (clade 2) contains Ascomycota that grow as yeast or pseudohyphae; the green clade (clade 3) and the purple clade (clade 4) are formed both by filamentous Basidiomycota and Mucoromycotina; and the brown clade (clade 5) grouped Microsporidia and Chytridiomycota.

The genomic blocks (centered on ChspIV and comprised of 31 putative genes) for each species were divided according to the five clades identified and aligned. Each of these genomic blocks' alignments were named as SynA, B, C, D, and E, respectively, to clades 1 to 5 (Table S10).

**Figure 4:**
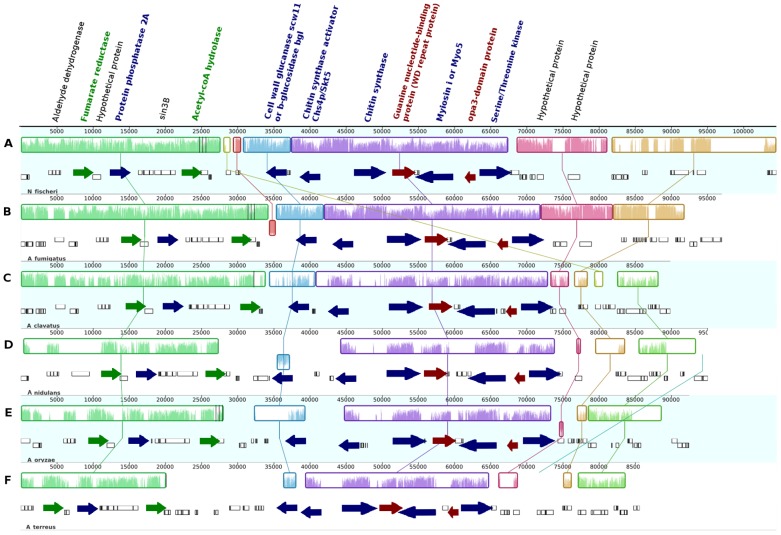
Syntenic analysis of SynA_1. Genomic blocks (Locally Collinear Blocks or LCBs, shown as colored boxes) from (A) *Neosartorya fischeri*, (B) *Aspergillus fumigatus*, (C) *A. clavatus*, (D *Emericella nidulans*, (E) *A. oryzae*, and (F) *A. terreus*. Vertical crisscrossing lines indicate orthology. Same color on gene names indicate functional relation. Arrow heads indicate direction of transcription.

Syntenic block SynA was further divided into four subgroups called SynA_1 to SynA_4 (Table S10). Since all members of subgroup SynA_1 belong to the genus Aspergillus (*A. fumigatus*, *A. clavatus*, *A. nidulans*, *A. oryzae*, *A. terreus*, and *Neosartoria fischeri* [Anamorph: *A. fischerianus*]) whose genomes are markedly syntenic [41], [42], its blocks show high conservation of gene order between each species (Figure 4). Orthologs across the six species for 15 of the 31 genes were identified. Besides the Chsp itself, at least five of these orthologous share a possible functional relationship (Figure S10): a chitin synthase activator, a class V (or class i) myosin, a serine/threonine kinase, a type 2A protein phosphatase PP2A (with a WD40 domain), and a cell wall glucanase scw11 (or beta-glucosidase *bgl*2). The chitin synthase activator located in this syntenic block shares its highest identity to Skt5p from *Coccidioides posadasii*, an ortholog of *S. cerevisiae*'s Chs4p/Skt5, a post-translational regulator of the Chs3p complex during vegetative growth [43].

It is remarkable the fact that the Chsp activator and the ChspIV gene are adjacent to each other on the genomic block and transcribed in a divergent orientation (Figure 4) in a “head-to-head” gene organization typical of fungal secondary metabolism gene clusters [44] or functionally related genes that show correlated transcriptional regulation [45]. Fungal hyphae cells, which grow on a polar-oriented way, require long-distance transport mechanisms and myosins participate in this process. It has been shown that Myosin-V accumulates in the apex of *Ustilago maydis* hyphae where vesicles cluster to form the fungal Spitzenkörper [46]. Class V Chsp contain a “Myosin Motor Domain” at their N-terminus (and are therefore also considered to be a class 17 myosin) which is used to transport it into the plasma membrane by binding to actin cables. Conversely, class IV Chsp lack this domain and need therefore to be transported by Myosin-V-containing vesicles [47]. Class V myosins have to be activated by phosphorylation at specific and highly conserved serine or threonine residues on its heavy chain. The kinase responsible for its activation is a member of the PAKS/STE201 family of serine-threonine protein kinases (such as the one found on this putative gene cluster) [48], [49]. On the other hand, type 2A protein phosphatases (PP2A) specifically dephosphorylate serine/threonine residues acting in opposition to the PAKS/STE201 kinase, as to regulate the activity of the class V myosin. While chitin synthases are involved in building up the cell wall, glucanases (such as scw11/BGL2) act antagonistically, degrading it and allowing cell growth [50].

**Figure 5:**
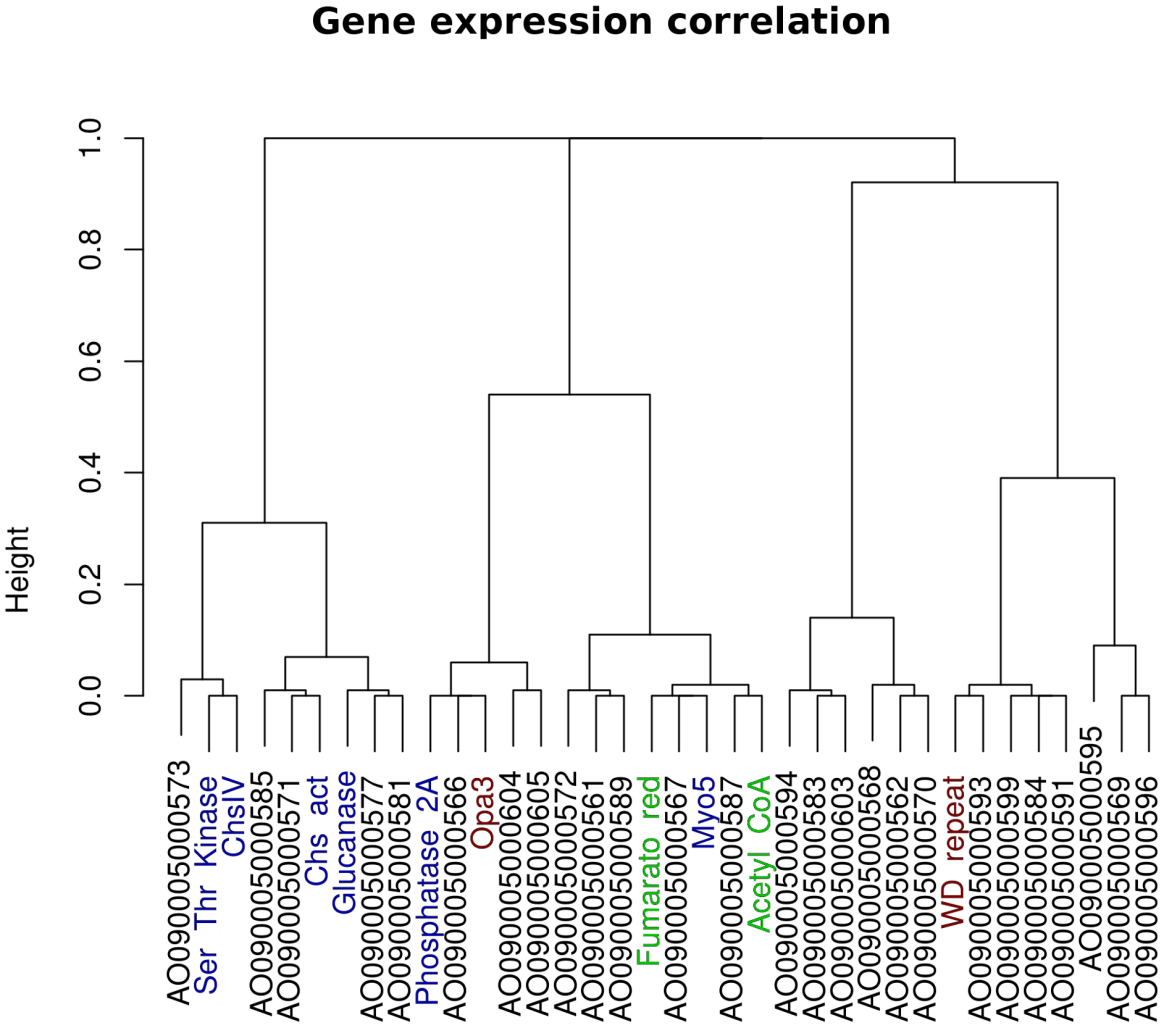
Gene expression correlation for putative cell-wall metabolism clutster. Hierarchical cluster dendrogram showing the correlations among gene expression for members of the putative cell-wall metabolism gene cluster on *Aspergillus oryzae* (NCBI GEO GSE9298) [37]. 36 probes (genes) were analyzed: the putative ChspIV plus 15 upstream and 20 downstream. Correlations are based on a dissimilarity measure of (1-*r* ^2^) in which correlation values are assigned “distance” values ranging from 0.0 (completely correlated, *r* ^2^ = 1) to 1.0 (completely uncorrelated, *r* ^2^ = 0). The y-axis represents the height or distance between the gene groups divided at that point.

An acetylCoA hydrolase and a fumarate reductase were also found in the putative syntenic block. Both of these enzymes are involved in mitochondrial carbohydrate metabolism for aerobic and anaerobic pathways [51]. Both of these genes are found at one of the boundaries of the putative cluster and may actually be outside of it. Finally, a guanine nucleotide-binding protein (wd repeat protein) and an opa3-domain protein were conserved across the members of the SynA_1 block. The guanine nucleotide-binding protein is similar to Asc1p, an ortholog of a protein associated to the 40S ribosomal subunit and involved on repression of gene expression [52]. The opa3-domain protein does not have a known function. Similar conservation is seen on the rest of SynA blocks (high conservation between orthologs of the respective block). Extending the orthology relation between the four SynA blocks there is, in varying degrees, conservation of genes which are related to cell wall metabolism. The chitin synthase activator is also found in the members of blocks SynA_2 (*F. graminearum*, *F. oxysporum*, and *F. verticilloides*) and SynA_3 (*C. globosum*, *N. crassa* and *P. anserina*). The cell wall glucanase can also be found in the SynA_3 and SynA_4 (*Botrytis cinerea* and *Sclerotinia sclerotiorum*) blocks (Figure S11). A correlation analysis of gene expression data for the putative gene cluster in *A. oryzae* (NCBI GEO GSE9298, grown on glucose [37]) shows an association between the transcription levels for ChspIV, the chitin synthase activator, the glucanase and the Ser/Thr kinase (Figure 5). All in all, this arrangement of genes related to the cell-wall metabolism or their regulation, strongly suggests this is a functional gene cluster, the first ever reported of its kind.

## Supporting Information

Figure S1 **Domains in putative Chsp (RE search).** Domains detected by InterProScan (PfamA) for the 369 putative Chsp sequences recovered by the RE search. Same color scheme as Figure S3.
(TIFF)

Figure S2 **Domains in putative Chsp (HMM search).** Domains detected by InterProScan (PfamA) for the 22 putative Chsp sequences recovered by the HMM search. Same color scheme as Figure S3.
(TIFF)

Figure S3 **Domain architecture for the seven Chsp classes.** Sizes of average sequences are roughly to scale. Drawn from data from this study and [6].
(TIFF)

Figure S4 **Tree inferred from conserved motifs (CON1S) with the NJ method.** Evolutionary model and parameters on methodology. Bootstrap consensus tree inferred from 1000 replicates, to represent the evolutionary history of the taxa analyzed.
(TIFF)

Figure S5 **Tree inferred from conserved motifs (CON1S) with the ME method.** Evolutionary model and parameters on methodology.
(TIFF)

Figure S6 **Tree inferred from conserved motifs (CON1S) with the Bayesian method.** The tree that had the highest posterior probability LnL in the approximate tree found by the numerical method of Markov Chain Monte Carlo with a chain length of 1,100,000.
(TIFF)

Figure S7 **Tree inferred from conserved motifs (CON1S) with the MP method.** Bootstrap consensus tree, inferred from 1000 replicates, to represent the evolutionary history of the taxa analyzed.
(TIFF)

Figure S8 **Chsp full protein sequences tree, inferred with the NJ method.** Bootstrap consensus tree, inferred from 10,000 replicates, to represent the evolutionary history of the taxa analyzed.
(TIFF)

Figure S9 **Chsp full protein sequences tree, inferred with the Bayesian method.** The tree that had the highest posterior probability LnL in the approximate tree found by the numerical method of Markov Chain Monte Carlo with a chain length of 1,000,000 was noted.
(TIFF)

Figure S10 **Gene Ontology (GO) terms hierarchy for biological process.** The member genes of the putative cell-wall metabolism gene cluster are associated to their predicted GO terms.
(TIFF)

Figure S11 **Orthology across all synthenic blocks.** Orthology, indicated by vertical crisscrossing lines, across all members of the syntenic blocks SynA (SynA_1 to SynA_4), SynB, and SynC; LCBs shown as colored boxes.
(TIFF)

Table S1 **Genomes and protein models used in this study and corresponding databases.**
(XLSX)

Table S2 **Chsp located by RE search.** 369 putative Chsp located in 54 genomes, using the regular expression method.
(XLSX)

Table S3 **49 Chsp from Uniprot database specifying species and accession.**
(XLSX)

Table S4 **34 Chsp from Uniprot database specifying name, accession, and class.**
(XLSX)

Table S5 **List of the 89 putative ChspIV used for synteny analysis.**
(XLSX)

Table S6 **Chsp located by HMM search.** 22 putative Chsp located on 54 genomes using the HMM method. Color code: (Red), truncated CON1S regions; (Yellow), single non-conserved change on CON1S; (Green), two highly conserved changes on EDRXL sequence; (Blue), CS2 domain detected by InterproScan; (Gray), CS1-CSN domain detected by InterProScan.
(XLSX)

Table S7 **Putative Chsp clade groupings by five phylogenetic methods, using only the canonical conserved motifs.** The squares represent monophyletic clade, triangles paraphyletic groupings, and circles polytomies.
(XLSX)

Table S8 **Putative Chsp clade groupings by two phylogenetic methods, using full sequences.** The squares represent clades and circles monophyletic polytomies.
(XLSX)

Table S9 **Putative Chsp classification by Phylogenetic inferences I and II.**
(XLSX)

Table S10 **Species groupings by syntenic genomic groups.**
(XLSX)

File S1 **Regular expression.** Regular expression based on the CON1S region and coded in a script written in the Perl programming language.
(PL) Click here for additional data file.
