# Supplementary figures and images for: Large-Scale Phylogenetic Classification of Fungal Chitin Synthases and Identification of a Putative Cell-Wall Metabolism Gene Cluster in Aspergillus Genomes
Source: PLoS One. 2014 Aug 22;9(8):e104920. doi: 10.1371/journal.pone.0104920 (PMC4141765; doi:10.1371/journal.pone.0104920)

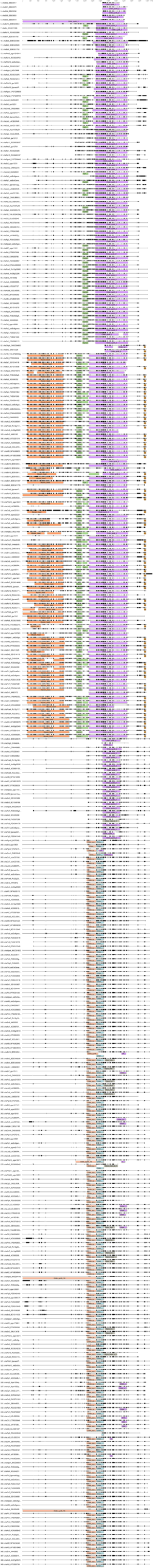

Supplement: Figure S1 — Domains in putative Chsp (RE search). Domains detected by InterProScan (PfamA) for the 369 putative Chsp sequences recovered by the RE search. Same color scheme as Figure S3. (TIFF) [file pone.0104920.s001.tiff]

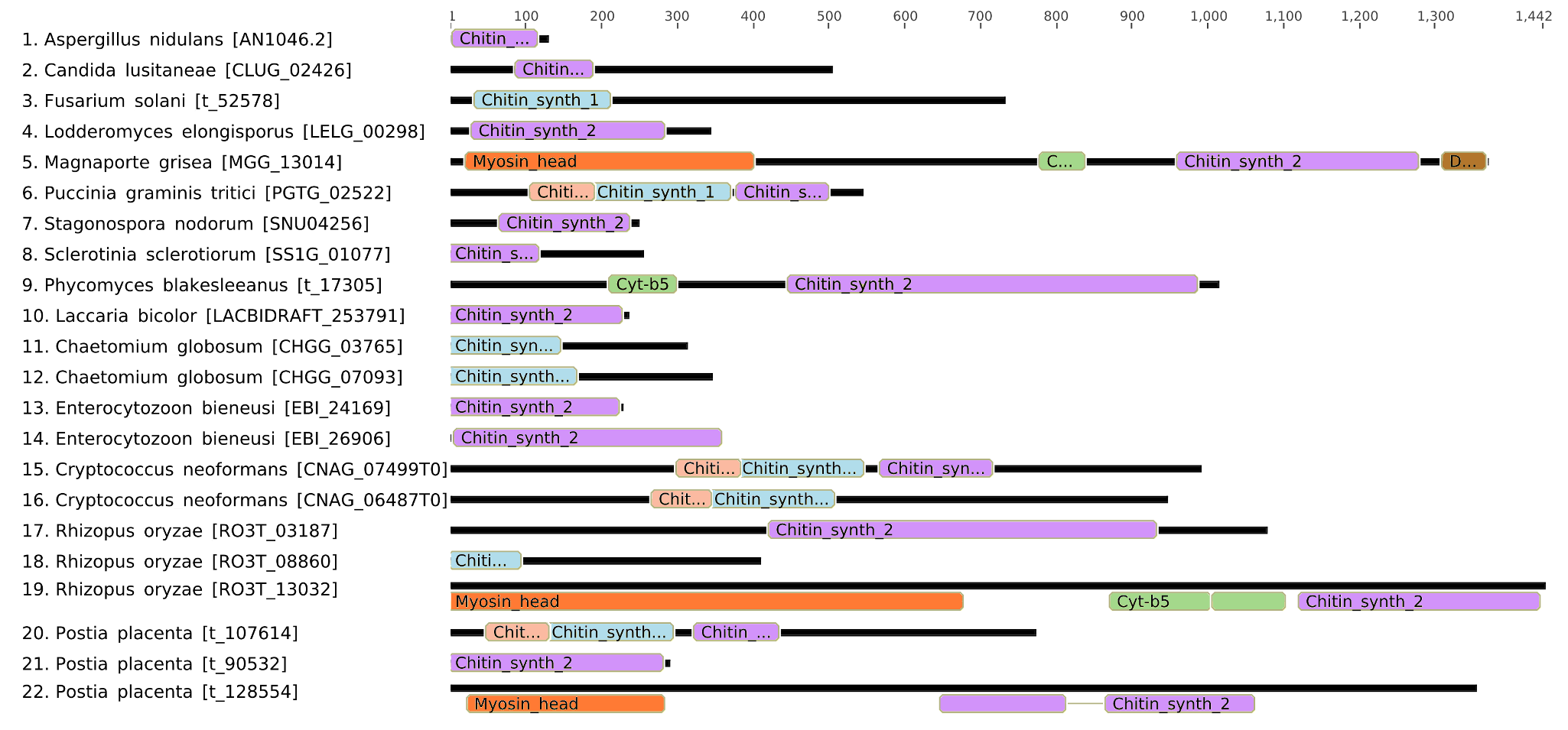

Supplement: Figure S2 — Domains in putative Chsp (HMM search). Domains detected by InterProScan (PfamA) for the 22 putative Chsp sequences recovered by the HMM search. Same color scheme as Figure S3. (TIFF) [file pone.0104920.s002.tiff]

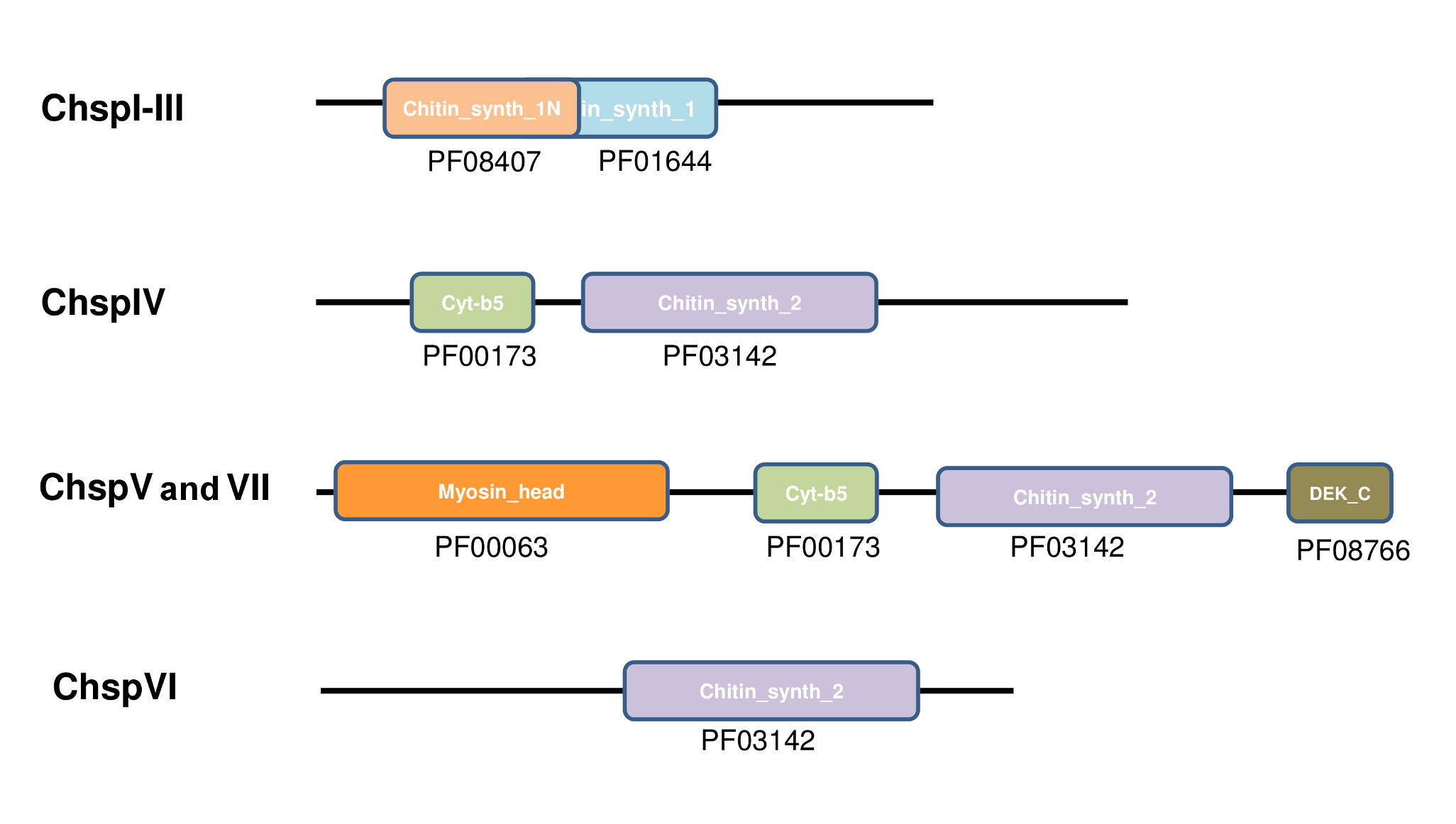

Supplement: Figure S3 — Domain architecture for the seven Chsp classes. Sizes of average sequences are roughly to scale. Drawn from data from this study and [6]. (TIFF) [file pone.0104920.s003.tiff]

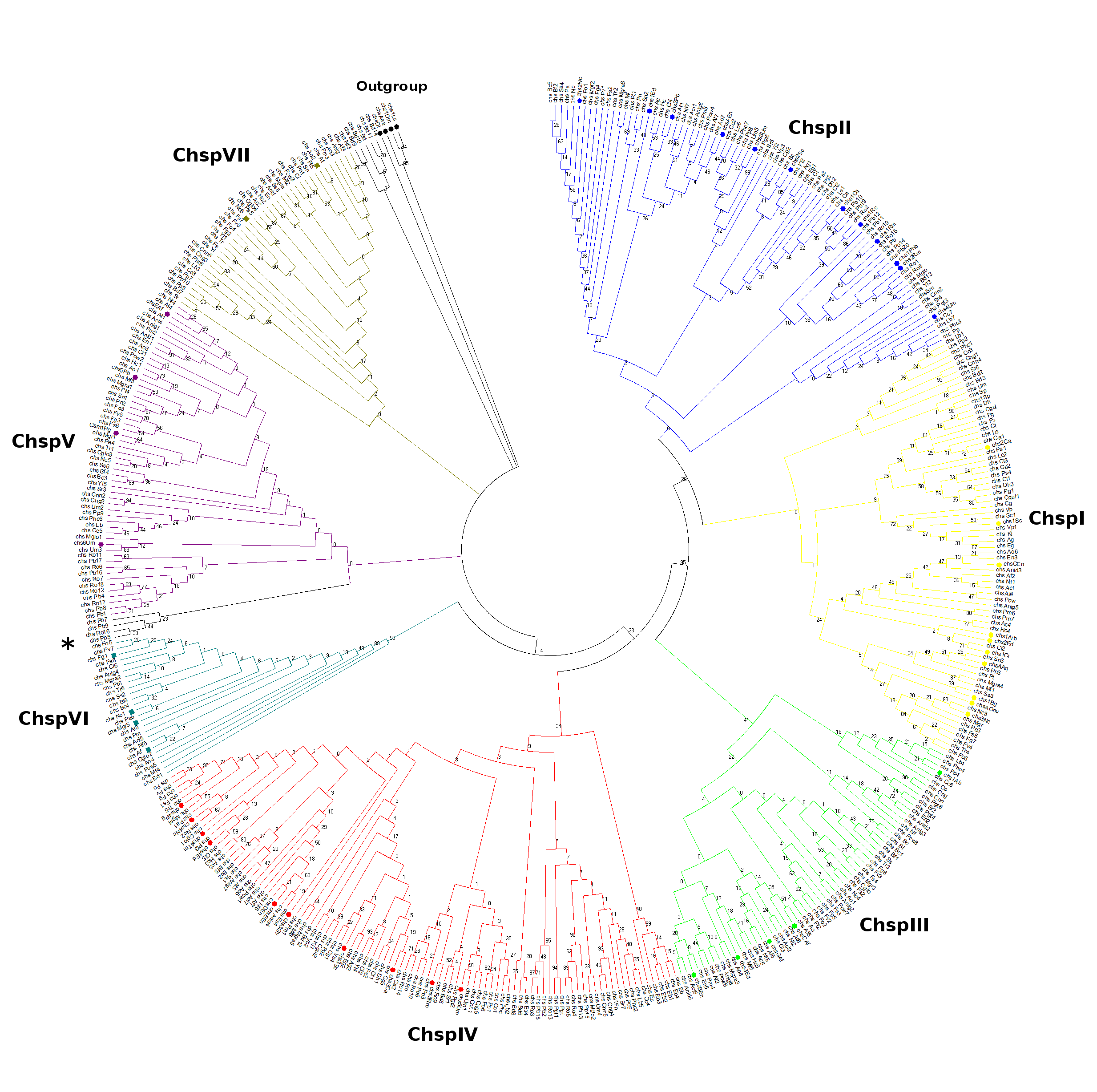

Supplement: Figure S4 — Tree inferred from conserved motifs (CON1S) with the NJ method. Evolutionary model and parameters on methodology. Bootstrap consensus tree inferred from 1000 replicates, to represent the evolutionary history of the taxa analyzed. (TIFF) [file pone.0104920.s004.tiff]

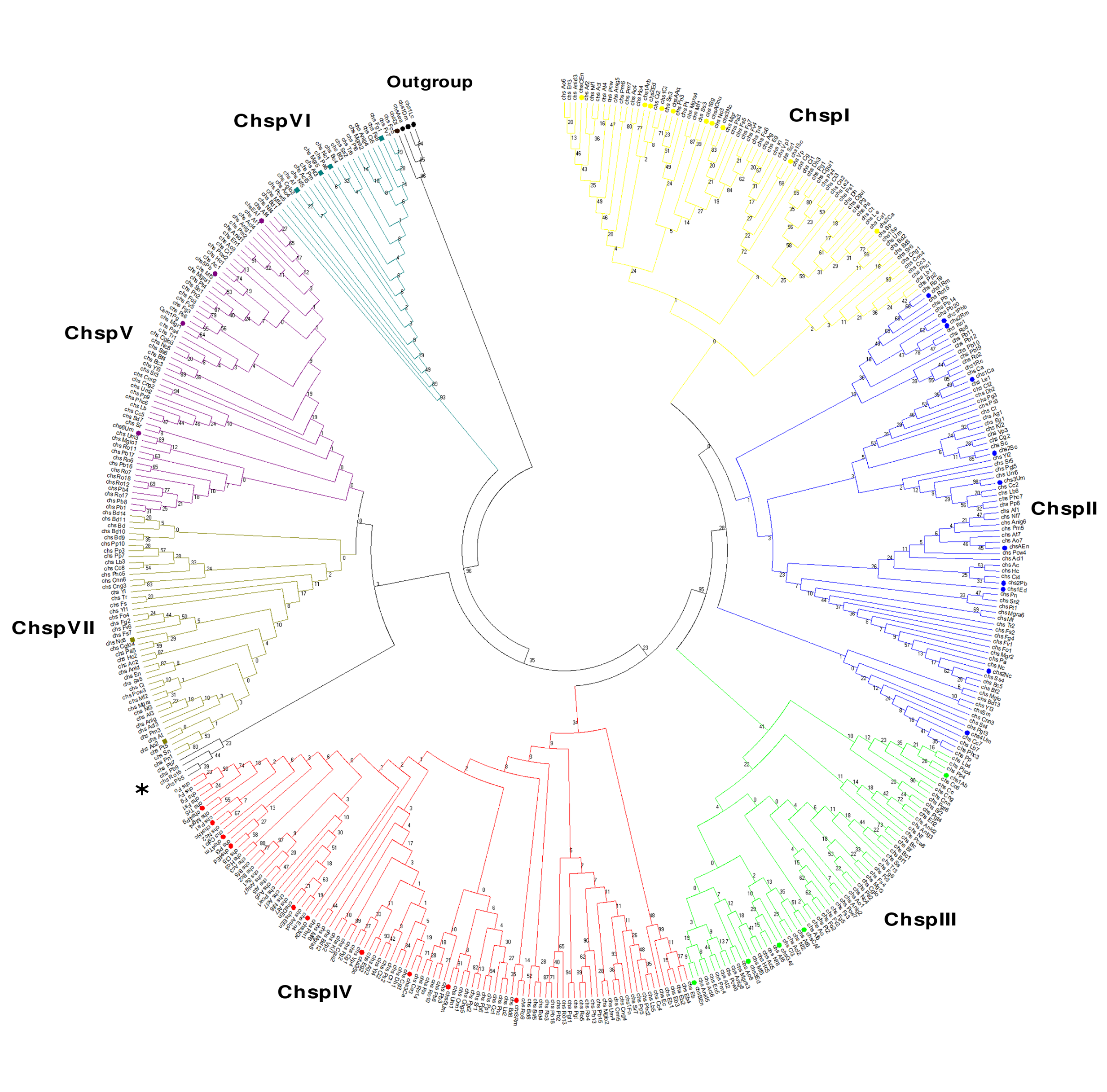

Supplement: Figure S5 — Tree inferred from conserved motifs (CON1S) with the ME method. Evolutionary model and parameters on methodology. (TIFF) [file pone.0104920.s005.tiff]

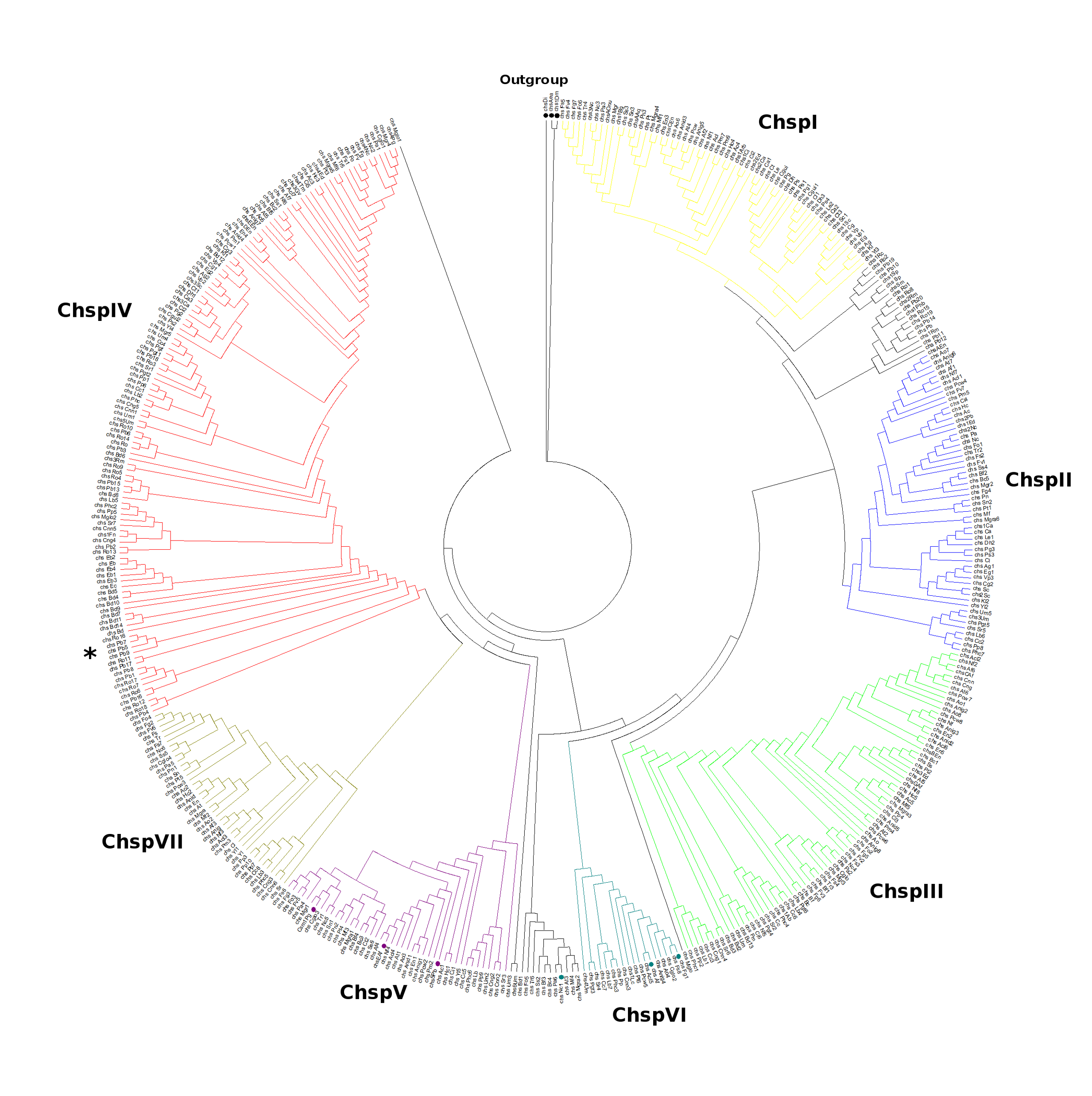

Supplement: Figure S6 — Tree inferred from conserved motifs (CON1S) with the Bayesian method. The tree that had the highest posterior probability LnL in the approximate tree found by the numerical method of Markov Chain Monte Carlo with a chain length of 1,100,000. (TIFF) [file pone.0104920.s006.tiff]

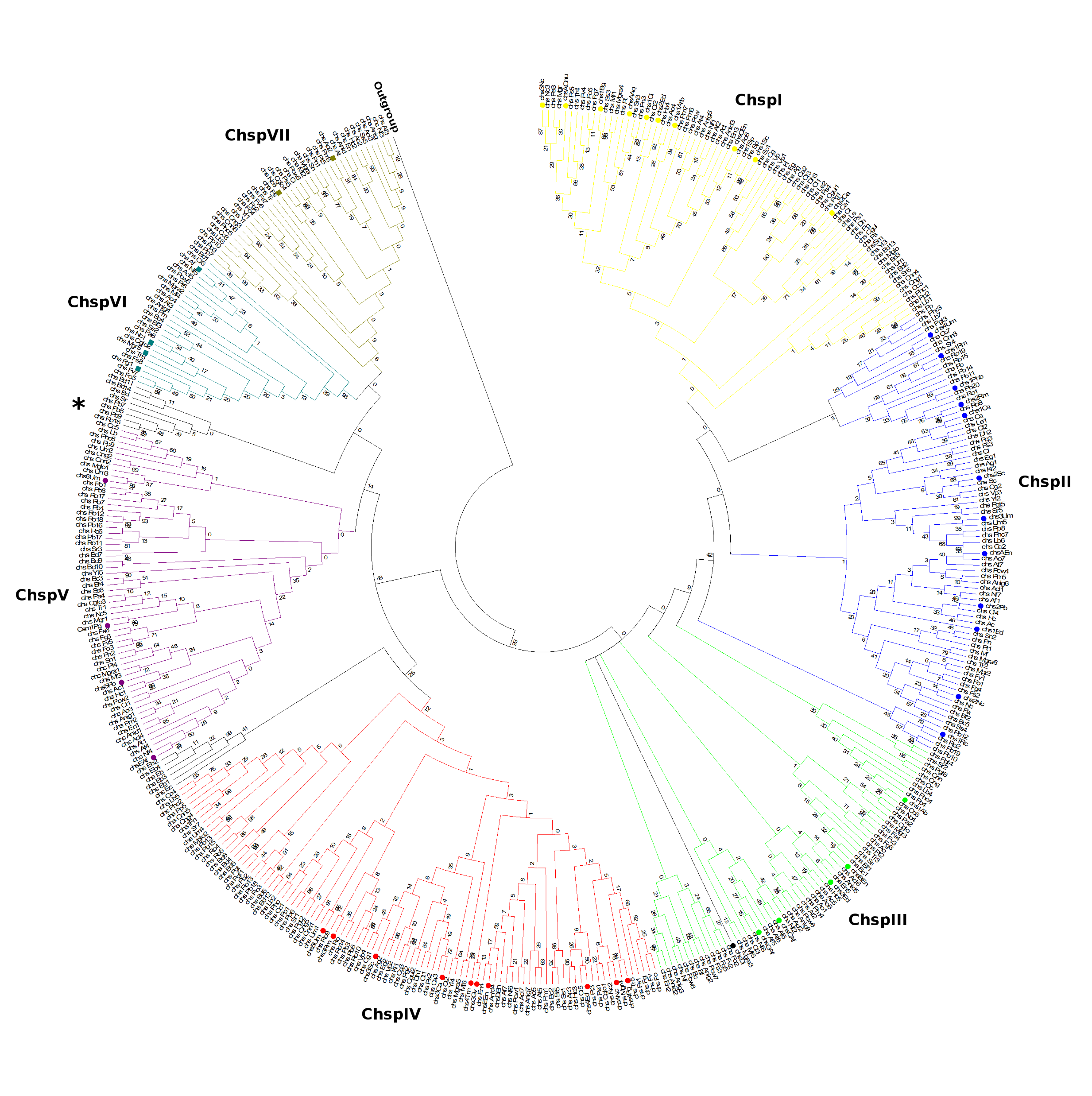

Supplement: Figure S7 — Tree inferred from conserved motifs (CON1S) with the MP method. Bootstrap consensus tree, inferred from 1000 replicates, to represent the evolutionary history of the taxa analyzed. (TIFF) [file pone.0104920.s007.tiff]

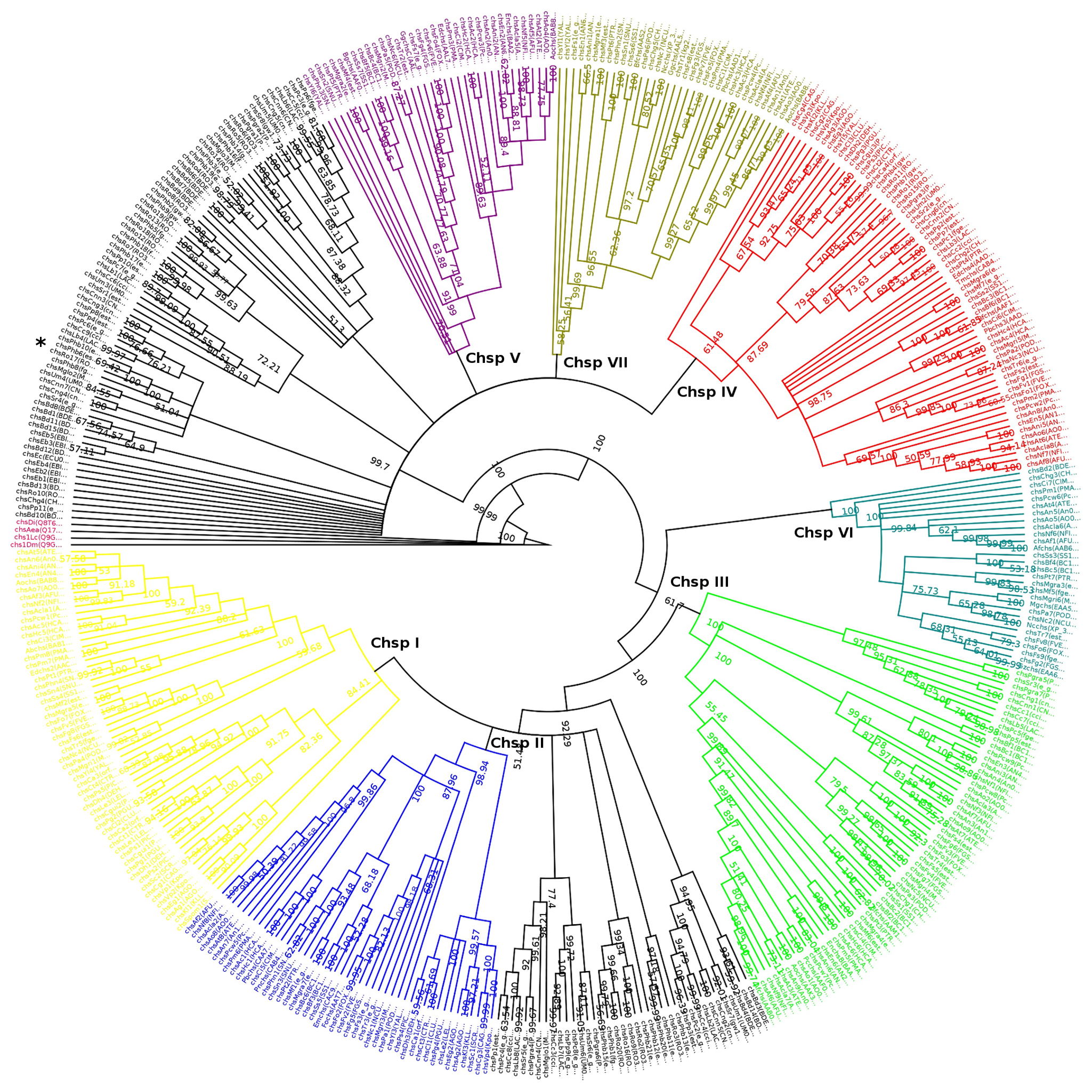

Supplement: Figure S8 — Chsp full protein sequences tree, inferred with the NJ method. Bootstrap consensus tree, inferred from 10,000 replicates, to represent the evolutionary history of the taxa analyzed. (TIFF) [file pone.0104920.s008.tiff]

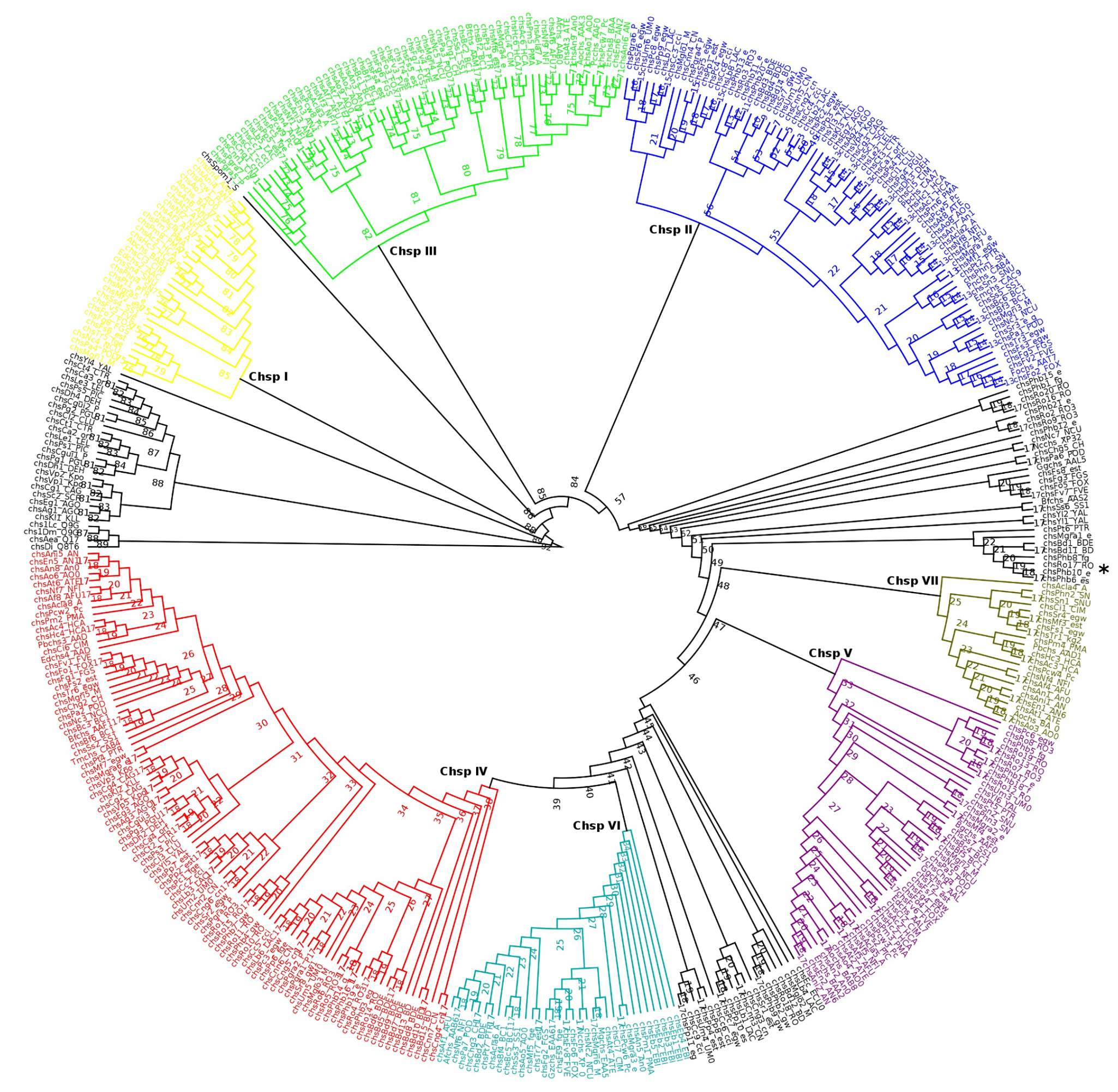

Supplement: Figure S9 — Chsp full protein sequences tree, inferred with the Bayesian method. The tree that had the highest posterior probability LnL in the approximate tree found by the numerical method of Markov Chain Monte Carlo with a chain length of 1,000,000 was noted. (TIFF) [file pone.0104920.s009.tiff]

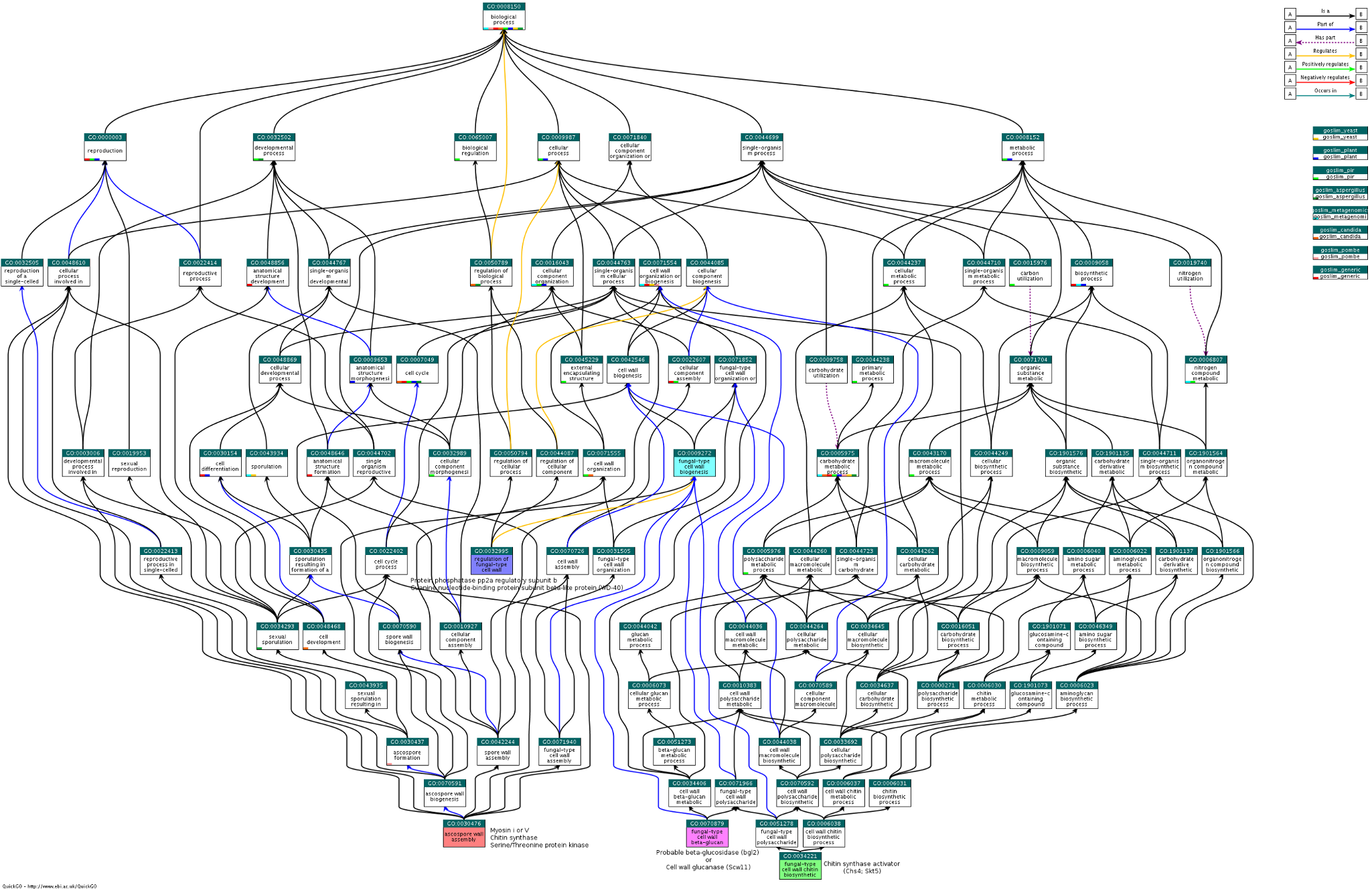

Supplement: Figure S10 — Gene Ontology (GO) terms hierarchy for biological process. The member genes of the putative cell-wall metabolism gene cluster are associated to their predicted GO terms. (TIFF) [file pone.0104920.s010.tiff]

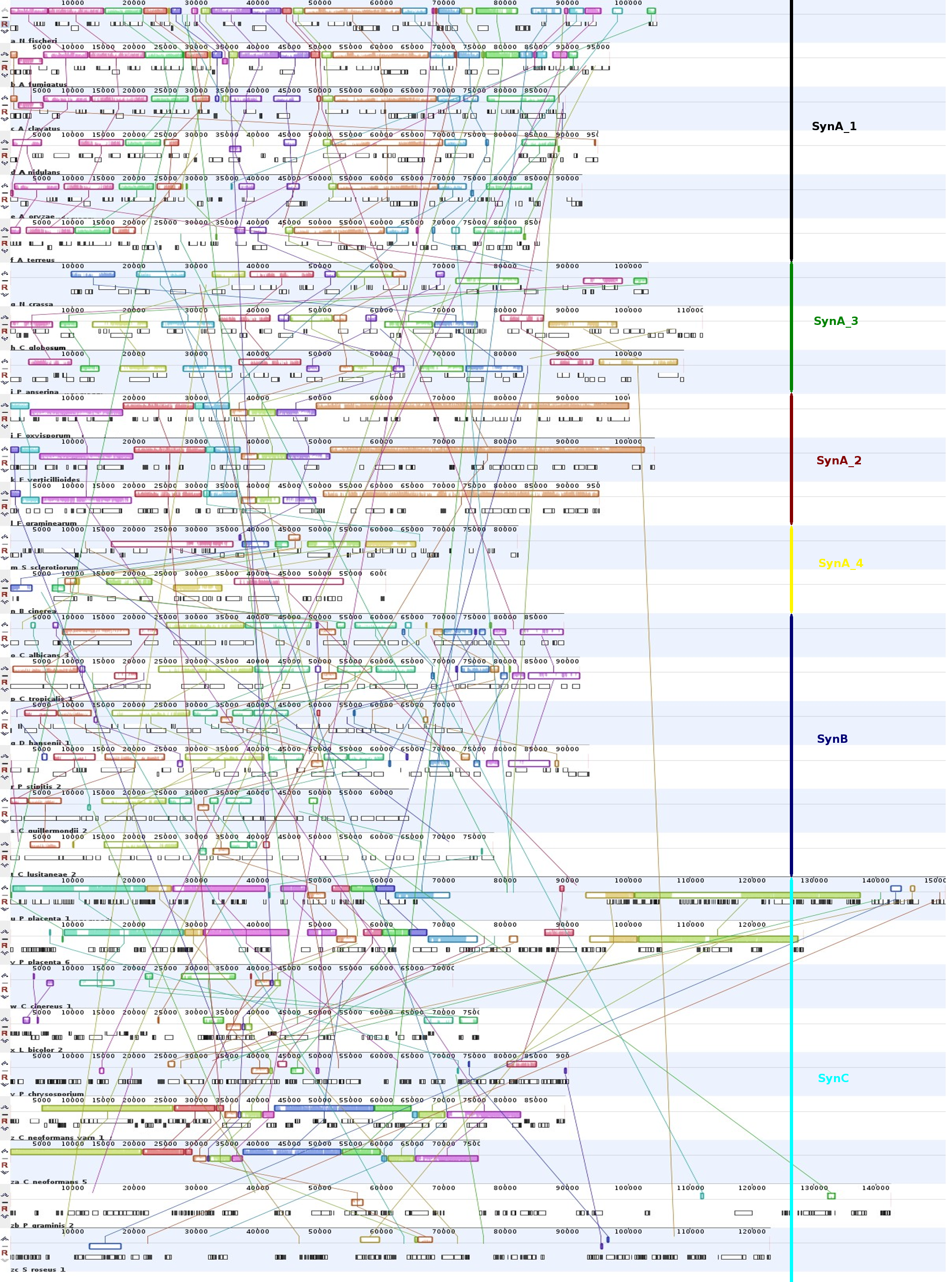

Supplement: Figure S11 — Orthology across all synthenic blocks. Orthology, indicated by vertical crisscrossing lines, across all members of the syntenic blocks SynA (SynA_1 to SynA_4), SynB, and SynC; LCBs shown as colored boxes. (TIFF) [file pone.0104920.s011.tiff]
